# Quantitative and automatic plan-of-the-day assessment to facilitate adaptive radiotherapy in cervical cancer

**DOI:** 10.1088/1361-6560/ade197

**Published:** 2025-06-23

**Authors:** Sarah A Mason, Lei Wang, Sophie E Alexander, Susan Lalondrelle, Helen McNair, Emma J Harris

**Affiliations:** 1 The Institute of Cancer Research, London, United Kingdom; 2 The Royal Marsden NHS Trust, London, United Kingdom

**Keywords:** plan of the day, adaptive radiotherapy, cervical cancer, cone-beam CT, artificial intelligence, female pelvic segmentation model

## Abstract

*Objective.* To facilitate implementation of plan-of-the-day (POTD) selection for treating locally advanced cervical cancer (LACC), we developed a POTD assessment tool for CBCT-guided radiotherapy (RT). A female pelvis segmentation model (U-Seg3) is combined with a novel quantitative standard operating procedure (qSOP) to identify optimal and acceptable plans. *Approach.* The planning CT[i], corresponding structure set[ii], and manually contoured CBCTs[iii] (*n* = 226) from 39 LACC patients treated with POTD (*n* = 11) or non-adaptive RT (*n* = 28) were used to develop U-Seg3, an algorithm incorporating deep-learning and deformable image registration techniques to segment the low-risk clinical target volume (LR-CTV), high-risk CTV (HR-CTV), bladder, rectum, and bowel bag. A single-channel input model (iii only, U-Seg1) was also developed. Contoured CBCTs from the POTD patients were (a) reserved for U-Seg3 validation/testing, (b) audited to determine optimal and acceptable plans, and (c) used to empirically derive a qSOP that maximised classification accuracy. *Main results.* The median (interquartile range) dice similarity coefficient (DSC) between manual and U-Seg3 contours was 0.83 [0.80], 0.78 [0.13], 0.94 [0.05], 0.86 [0.09], and 0.90 [0.05] for the LR-CTV, HR-CTV, bladder, rectum, and bowel. These were significantly higher than U-Seg1 in all structures but bladder. The qSOP classified plans as acceptable if they met target coverage thresholds (LR-CTV $\unicode{x2A7E}$ 99%, HR-CTV $\unicode{x2A7E}$ 99.8%), with lower LR-CTV coverage ($\unicode{x2A7E}$95%) sometimes allowed. The acceptable plan minimizing bowel irradiation was considered optimal unless substantial bladder sparing could be achieved. With U-Seg3 embedded in the qSOP, optimal and acceptable plans were identified in 46/60 and 57/60 cases. *Significance.* U-Seg3 outperforms U-Seg1 and all known CBCT-based segmentation models of the female pelvis both in terms of scope and accuracy (median DSC improvement ranging from 0.03–0.06). The tool combining U-Seg3 and the qSOP identifies optimal plans with equivalent accuracy as two observers. In an implementation strategy whereby this tool serves as the second observer, plan selection confidence and decision-making time could be improved whilst simultaneously reducing the required number of POTD-trained radiographers by 50%.

## 1. Introduction

External beam radiotherapy (EBRT) is part of curative treatment for locally advanced cervical cancer (LACC), where the clinical target volume (LR-CTV) includes the cervix, uterus, upper vagina and parametria (Lim *et al* 2011). The LR-CTV undergoes large amounts of interfractional motion and deformation due to physiological factors such as bladder filling, rectal filling, and tumour regression, making it challenging to accurately target with EBRT (Taylor and Powell 2008, Jadon *et al* 2014). To compensate for this motion, the traditional and most widely used approach in RT is to deliver plans with a large CTV-to-PTV (planning target volume) margin to increase the likelihood of adequate target coverage (Wang *et al* 2023). However, this comes at the cost of including large volumes of healthy tissues in the high dose region which is correlated with increased rates and severity of radiation induced toxicity for these patients (Yeung *et al* 2020, Seppenwoolde *et al* 2021, Ghosh *et al* 2024). Indeed, a substantial number of patients (18.4%) are expected to experience grade 3 or higher morbidity within 5 years of treatment with conventional EBRT approaches (Pötter *et al* 2021).

Advances in image-guidance technologies have enabled the development of ‘adaptive radiotherapy’, whereby a RT plan based on the patient’s anatomy at the time of treatment can be delivered. This allows the use of smaller PTVs that conform to the shape of the LR-CTV, thus producing more optimal dose distributions. Two examples of adaptive RT include plan-of-the-day (POTD) selection and online adaptive RT (oART).

POTD selection involves (1) generating a library of plans in advance of RT treatment based on the anticipated motion of the LR-CTV, (2) acquiring a daily image once the patient has been set up in the treatment position to enable visualisation of the CTVs and OARS, and (3) selecting the most appropriate plan based on the internal anatomy at the time of treatment (Seppenwoolde *et al* 2016, Nováková *et al* 2017, Wang *et al* 2023, Ghosh *et al* 2024, Reijtenbagh *et al* 2024). It has been demonstrated that the POTD approach for LACC patients provides significant dosimetric benefit over non-adaptive RT in terms of OAR sparing whilst maintaining or even improving upon LR-CTV coverage (van de Schoot *et al* 2017, Buschmann *et al* 2018, Wang *et al* 2023, Reijtenbagh *et al* 2024). Another appeal of POTD selection is that it can be integrated into pre-existing workflows on conventional linacs, and thus presents an exciting opportunity to make adaptive RT widely accessible.

An alternative strategy is oART, whereby a new treatment plan is generated at each fraction with the aim of maximising the conformity of the high dose region to the CTV. Although positive dosimetric outcomes using online replanning to treat LACC patients have been reported (Ding *et al* 2023, Shelley *et al* 2023), this technique is not yet widely used or available. oART is a resource intensive process requiring specialised delivery systems, relatively long treatment times (primarily due to contour generation and plan recalculation/reoptimisation), and a multi-disciplinary team of radiation therapists, radiation oncologists and physicists on-set. An additional vendor-specific challenge is the fact that the primary and elective targets for node positive patients cannot simultaneously fit within the field length of the Elekta Unity MR-Linac (Portelance *et al* 2021, Shelley *et al* 2021), making them sub-optimal candidates for this oART platform. Although addressing the bottlenecks and limitations of online replanning is an active area of research with many promising solutions on the horizon (Branco *et al* 2023, Chuter *et al* 2023, Rayn *et al* 2024), it is unclear when these technologies will become more widely accessible.

Although POTD selection is clinically implemented to a greater extent than daily online replanning for LACC, there are still barriers that prevent it from being the standard of care despite the proven dosimetric benefit and institutional desire (Bertholet *et al* 2020). Indeed, it is estimated that only 6%–25% of centres (depending on the country) implement POTD selection for cervical cancer (Bertholet *et al* 2020, Reijtenbagh *et al* 2024). One barrier is the increased time and effort required to generate multiple plans when building the library: done manually, generating a 4-plan library for LACC treatment takes approximately double the time it would do to generate a single plan for non-adaptive RT (Buschmann *et al* 2018, Wang *et al* 2023). Commercially available fully automatic planning solutions such as iCycle may be used to alleviate this burden, and have indeed demonstrated good performance in LACC patients (Rhee *et al* 2020). There are also barriers associated with implementation: not only do institutions typically need to set up training and auditing programs to ensure the quality of plan selection, but there needs to be sufficient staff uptake to enable the best practice of having two radiation therapists on-set to agree on the optimal plan (McNair *et al* 2015, 2022, Alexander *et al* 2019, Webster *et al* 2021, Wang *et al* 2023). The latter is resource intensive and can disrupt the workflow on other units. The quality of the daily image is also a concern. Although CBCT imaging is the current image-guidance workhorse in modern RT departments, it suffers from relatively poor soft tissue contrast and image artefacts which can make it difficult and time consuming for human observers to discern soft tissue boundaries in the female pelvis (Heijkoop *et al* 2014). Even with well-established LACC POTD workflows in place, there is still discordance (ranging from 40%–14% depending on institution) between the plan chosen for delivery on-set and the optimal plan as identified in offline central reviews (Gobeli *et al* 2018, Alexander *et al* 2019).

Plan selection is a complex process requiring observers to simultaneously consider target coverage, OAR sparing, anticipated intrafraction motion, consequences of getting the patient off the couch in cases of suboptimal dosimetry, nodal coverage at previous fractions, frequency with which large-PTV ‘robust’ plan has been previously used, and adherence to institutional protocol regarding on-set decision-making. Without any quantitative information available, it is not only difficult to judge what plan is the best (if any) but is also difficult to encode into a standard operating procedure (SOP) that (a) is objective and (b) consistently reflects the true optimum. The plan selection SOP used at our institution at the outset of this study is shown in appendix A. This SOP uses language such as ‘best coverage’, ‘least bowel’, and ‘least bladder’, which is inherently qualitative or binary as the relative volumes of each structure are judged by eye.

Developing tools to automate pelvic organ segmentation and/or plan selection could help alleviate the time, subjectivity, and resource burden currently faced in the clinic. Several groups have proposed methods for automatically segmenting the uterus-cervix complex on CBCT images using atlas-based and AI-based techniques (Langerak *et al* 2014, Beekman *et al* 2022, Zhang *et al* 2022). Although some of these have been shown to have good agreement with manual contours, considering the uterus-cervix complex alone is not sufficient for determining the most optimal plan as (1) the whole LR-CTV is not segmented (i.e. the parametria are excluded) and (2) there is no provision for dealing with the likely scenario in which multiple plans provide full target coverage. To our knowledge, only Zhang *et al* (2022) have contoured both uterocervix-complex and OAR structures on CBCT in the female pelvis for the purpose of automatic POTD selection. We expand on their work to increase the clinical relevance of this approach by:

• Considering the complete LR-CTV (including the parametria) and high-risk CTV (HR-CTV). Although these structures cannot be directly visualized on CBCT, their position can be inferred from pre-treatment imaging and surrounding anatomy. As per RTOG guidelines, it is essential to include the parametria into the target volume to improve local control of disease (Lim *et al* 2011). The HR-CTV (i.e. the gross tumour and uninvolved cervix) should also be considered as it as important part of the decision-making process, particularly in cases when 100% LR-CTV coverage is not achievable with any plan in the library.
• Evaluate the potential benefit of including **patient-specific prior information** into the network to improve segmentation accuracy (1) overall and (2) in outlier cases where unique anatomies and/or image artefacts result in poor segmentation and therefore incorrect plan selection.
• Using OAR segmentations to guide target segmentation *and* directly inform optimal plan selection.
• Evaluate POTD selection accuracy using an actual clinical library of PTVs rather than overlap metrics between CBCT and planning CT uterus-cervix complex segmentations. Although one might assume that the PTV and plan generated from the CT image with the highest uterus-cervix complex segmentation overlap would be optimal, it fails to address cases where no plans are acceptable (e.g. the uterus-cervix complex is outside of the PTV), or when multiple cases are acceptable in terms of target coverage and the optimal plan is judged on OAR coverage.

To overcome the limitations of previous attempts, we have developed ‘U-Seg3’ which automatically segments the whole LR-CTV, HR-CTV, bladder, rectum, and bowel bag on CBCT images for LACC patients. U-Seg3 incorporates patient-specific prior knowledge obtained during planning into a two-step algorithm using a combination of deep-learning and deformable image registration (DIR) techniques. The prospect of having these structures segmented automatically on-set enables the consideration of *quantitative* POTD selection SOPs, where PTV coverage thresholds can be defined for individual structures. To this end, we have identified a set of optimal target and OAR coverage constraints based on plan selections performed at our institution to form a decision boundary between acceptable and unacceptable plans: this is henceforth referred to as the ‘quantitative SOP’ (qSOP). We demonstrate that U-Seg3 not only outperforms all previous methods in terms of segmentation accuracy, but also has the potential to revamp POTD selection by (a) making it fully automatic and (b) enabling the use of qSOPs, which could ultimately lower the resource burden and subjectivity of this adaptive RT approach.

## 2. Materials and methods

### 2.1. RT planning and treatment

Data from 39 LACC patients treated with 25 fractions of EBRT, weekly cisplatin chemotherapy, and brachytherapy at our institution were included in this study. The FIGO cancer stage of this patient cohort ranged from 1B2–4B. All patients gave written informed consent for their anonymised data to be used for research.

#### 2.1.1. Pre-treatment imaging and planning

Planning CT scans of each patient were acquired with two different bladder statuses:

(i) Empty bladder CT: Acquired immediately after the patient voided her bladder.
(ii) Full bladder (FB) CT: Acquired with IV contrast an hour later after the patient drank 350 ml of water. This scan was used for RT plan calculation and available for reference on-set during POTD selection.

Patients also received a planning magnetic resonance image (MRI), where an intermediate bladder filling was achieved by voiding the bladder and subsequently drinking 350 ml of water 30 min prior to scanning. The patient was in the supine position for all imaging procedures.

Planning CT and MR images were imported into RayStation (Version 12-R, RaySearch Laboratories AB, Stockholm, Sweden) for contouring and treatment planning. A radiation oncologist contoured the LR-CTV, bladder, rectum, sigmoid, bowel bag, and involved lymph nodes following the EMBRACE-II protocol on all images (Pötter *et al* 2018). The HR-CTV was additionally contoured on the planning MRI, and propagated to the planning CT via RayStation’s hybrid DIR algorithm using the LR-CTV as a controlling ROI. As a precautionary measure, the deformed HR-CTV was then expanded by 10 mm superiorly and inferiorly within the LR-CTV to compensate for potential decreases in HR-CTV extent (i.e. compensating for shrinkage that might occur when deforming a larger LR-CTV to a smaller LR-CTV) or other positional uncertainties of the HR-CTV arising from the DIR process.

Given the three planning images over a range of bladder volumes, a radiation oncologist generated three sub-range internal target volumes (ITVs) based on the observed and anticipated range of motion of the LR-CTV, taking into account potential physiological changes unseen at planning such as tumour regression and variations in rectal filling as described by Wang *et al* (2024). The union of all three sub-range ITVs was uniformly expanded by 7 mm to form the ‘robust’ PTV. The three individual sub-range ITVs were uniformly expanded by 5 mm to form PTV1, PTV2, and PTV3, generally corresponding to small-bladder, mid-bladder, and full-bladder anatomies, respectively.

For the 11 patients treated from April 2022 onwards (when we clinically implemented POTD selection), a single or dual arc volumetric modulated arc therapy (VMAT) plan was generated for each of the four PTVs to build a four-plan library. The most appropriate plan from this library was delivered at each fraction as described in section 2.1.2. The 28 patients treated before April 2022 underwent non-adaptive RT, whereby a single VMAT plan was generated (with large CTV-PTV margins as in the Robust plan in the POTD approach) and delivered: no sub-range ITVs or corresponding small-PTV plans were created for these patients. Images from these patients were used exclusively to train the automatic segmentation component of U-Seg3, as described in section 2.3.1.

#### 2.1.2. Clinical POTD protocol

One hour before treatment, patients were asked to void their bladder and drink 350 ml of water to encourage a comfortably full bladder at the time of radiation delivery. After patient set-up, CBCT acquisition and bony anatomy registration (Alexander *et al* 2019), two radiation therapists certified in LACC POTD selection visually inspected the four PTVs available (PTV1, PTV2, PTV3, and PTV-Robust) overlaid with the daily CBCT and co-registered FB-CT using XVI (Elekta Ltd Stockholm Sweden, versions 5.0.4–5.0.6). Note, the PTVs as defined on the FB-CT were used as a surrogate for the volume of tissue receiving 45 Gy (Reijtenbagh *et al* 2024), and were treated as immutable structures. Each PTV was represented as a contour superimposed on the daily image where the positions of the LR-CTV, bowel, and bladder were judged by eye^3^3Rectal coverage was not considered at treatment as there was not much difference between the PTVs in the library at the rectum (see figure 5). However, rectal dose constraints were met at planning as per EMBRACE II guidelines.. The FB-CT and corresponding manual contours could be toggled on and off to help orient the observers with the patient’s anatomy to improve CBCT interpretation. The plan judged to have the most optimal PTV in terms of target and OAR coverage was delivered. As per standard practice, no recalculation or reoptimisation of the plan was performed given that dosimetric differences between the planned and delivered dose are assumed to be small [1%–2%] (Bortfeld *et al* 2004). The flowchart in appendix A outlines the initial SOP used to guide observers in the optimal plan selection. More information about the use of PTVs to select an optimal plan can be found in appendix B. In summary, the optimal plan should satisfy the following with regards to PTV coverage (in descending order of priority): full HR-CTV coverage, full LR-CTV coverage, minimal bowel coverage, and minimal bladder coverage. In cases where no plans were acceptable, the patient’s treatment was delayed for a later time that day after an appropriate intervention was performed to make the internal anatomy more suitable for the plans in the library (i.e. bladder filling, bowel emptying, etc). Offline imaging and replanning was performed to generate an updated plan library in the rare cases in which a patient consistently required intervention or the use of the robust plan.

### 2.2. Offline analyses

#### 2.2.1. CBCT contouring

The LR-CTV, bladder, rectum, and bowel bag on 5–11 CBCT images per patient were manually contoured by a radiation oncologist using RayStation. A semi-random subset of 5 images spanning the duration of treatment were initially contoured. Any remaining CBCTs that exhibited dissimilar anatomy from the initial contoured dataset (upon visual inspection) were additionally contoured (Wang *et al* 2024). This resulted in a total of 226 contoured CBCT images from 39 patients. Note: the parametria were included in the LR-CTV; as these are not directly visible on CBCT, this was achieved with careful and consistent technique by inferring their position from planning scans and CBCT-visible anatomical landmarks. The HR-CTV contour on the FB-CT was propagated to the contoured CBCTs via RayStation’s hybrid DIR algorithm using the LR-CTV as a controlling ROI.

#### 2.2.2. Optimal plan selection auditing

For the 11 POTD patients used in this study, all available daily CBCTs (*n* = 60) were assessed retrospectively by a radiation oncologist. A random subset of these (*n* = 10) were also assessed by an expert radiation therapist. Each observer independently identified the plan that would have been most optimal for delivery. The radiation oncologist also identified plans that were suboptimal, but still acceptable.

### 2.3. U-Seg algorithm development

**Figure 1.**
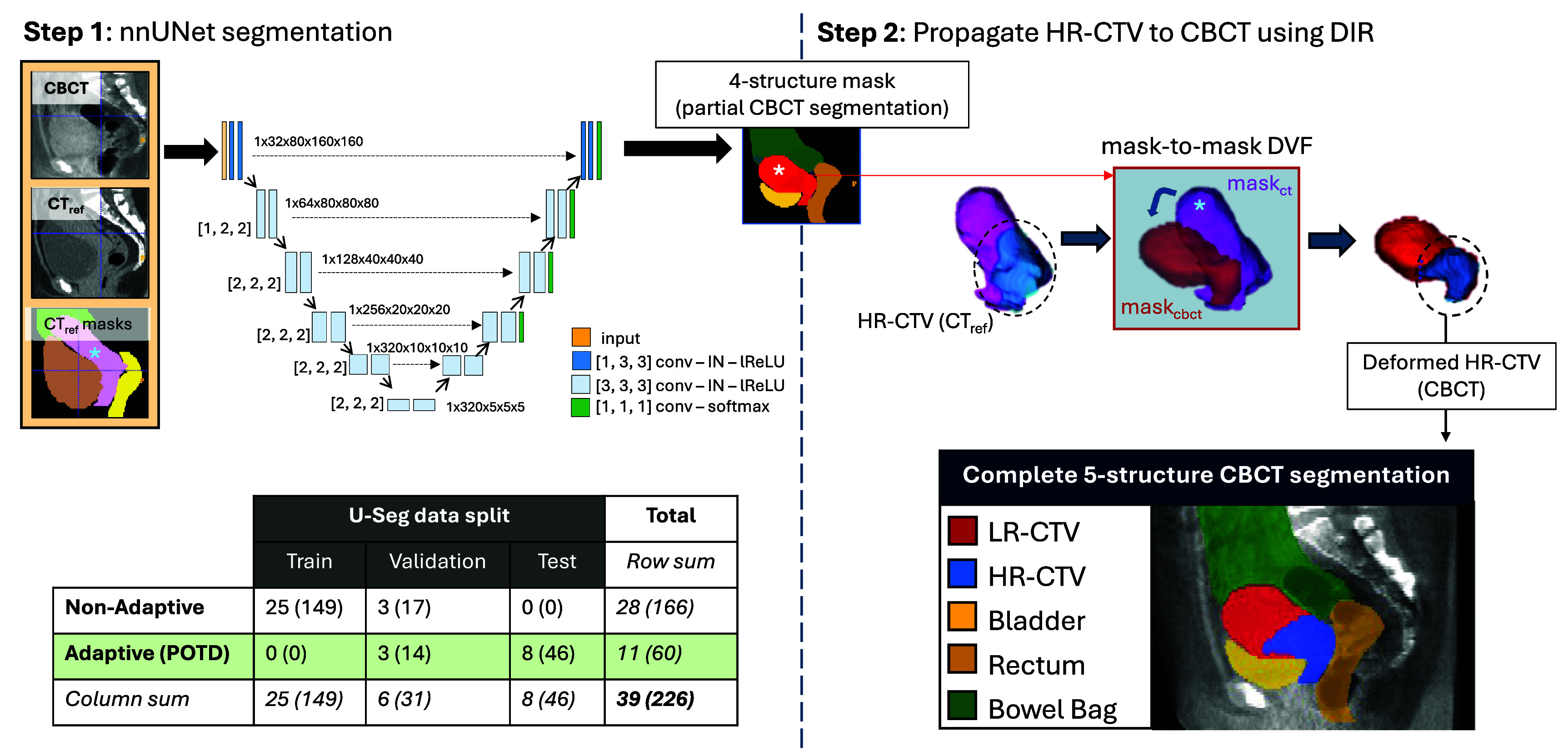
Schematic representation of U-Seg. Step 1 provides segmentation of the LR-CTV (red), bladder (yellow), rectum (orange), and bowel bag (green) on the daily CBCT. Possible inputs were: CBCT only [single channel] or CBCT, reference CT (CT_ref_) and CT_ref_ manual contours (CT_ref_ masks)[three-channel]. The nnUNet is represented by the rectangles, with each colour representing a specific block of operations as indicated by the legend ([X, X, X] = convolution kernel size, conv = convolution, IN = instance normalization, lReLU = leaky rectified linear unit). The numbers in brackets outside of the light blue rectangles indicate the stride, and the arrows with the dotted lines represent skip connections between encoder and decoder stages. The table indicates the number of patients (and corresponding number of manually contoured CBCTs in parentheses) in train, validation, and test sets for U-Seg algorithm development overall (*column sum*), and by treatment (non-adaptive row or adaptive row). Step 2 provides segmentation of the HR-CTV by propagating the HR-CTV contour onto the daily CBCT based on DIR between the reference LR-CTV mask (cyan asterisk on pink mask) and the nnUNet-generated LR-CTV mask (white asterisk on red mask). Combining the 4 structures segmented in Step 1 with the HR-CTV segmented in Step 2 provides the final segmentation of all 5 structures required for CBCT-based automatic POTD selection.

The U-Seg algorithm was designed to automatically segment the LR-CTV, bladder, rectum, bowel bag, and HR-CTV on daily CBCT images in a two-step process. Firstly, a 3D nnUNet was used to segment the LR-CTV, bladder, rectum, and bowel bag as described in section 2.3.1. Secondly, DIR was used to propagate the HR-CTV contour from the FB-CT image to the daily CBCT as described in section 2.3.2. The U-Seg algorithm is represented schematically in figure 1. Note: as per our clinical POTD protocol (see appendix A), the rectum is not considered as part of the plan selection process. The reason for segmenting it was to help overall observer interpretation of CBCT images and to guide the segmentation of surrounding structures. The rationale for segmenting the HR-CTV in a second step outside of the nnUNet is described in appendix C.

#### 2.3.1. Step 1: nnUNet development, training, and testing

We trained two versions of an nnUNet (Isensee *et al* 2021) to perform multi-organ segmentation on CBCT images of the female pelvis. The nnUNet is a state-of-the-art algorithm using the well-established U-Net architecture (Ronneberger *et al* 2015) designed specifically for medical image segmentation (Isensee *et al* 2021). The nnUNet automatically sets the tuneable network hyperparameters and number of encoder/decoder steps based on the ‘fingerprint’ of the training data along with performing all necessary preprocessing and data augmentation steps required for training. Given the impressive track record of the nnUNet in segmenting publicly available datasets (Isensee *et al* 2024) and the promising results reported by Zhang *et al*, we opted to accept the default network architecture and parameters (see figure 1 and appendix D) suggested by the algorithm. We used a single-fold, 3D full resolution configuration of the nnUNet to train (i) U-Seg1: a single-channel-input version and (ii) U-Seg3: a three-channel-input version to automatically segment the LR-CTV, bladder, bowel bag, and rectum on daily CBCT images.

(i) **U-Seg1 (single-channel version)**: The input to this network was the daily CBCT image alone. This version is in line with the method described by Zhang *et al* (2022) using our in-house data, and was used as a performance benchmark for segmentation accuracy.
(ii) **U-Seg3 (three-channel version)**: The 3 inputs to this network were: (1) the daily CBCT image, (2) the FB-CT image (rigidly registered to the CBCT image using the same translational shifts applied at treatment during the bone-match), and (3) the corresponding FB-CT manual segmentations of the LR-CTV, bladder, bowel bag, and rectum. Including this information from planning is the mechanism through which prior knowledge of patient-specific information was incorporated into the algorithm. We emphasize that there were no DIRs performed between the reference CT and daily CBCT, nor was there any requirement for image/anatomical similarity.

Aside from the number of input channels, all other parameters in U-Seg1 and U-Seg3 were held constant. Details of the specific network architecture is shown in figure 1. Figure 1 also demonstrates how the patients were divided into training, validation, and test sets. Note that none of the 11 POTD patients were used to train the nnUNet; this was done intentionally to preserve the integrity of analyses dealing with automatic POTD selection accuracy. Additional network parameters are given in appendix D.

#### 2.3.2. Step 2: segmentation of the HR-CTV

The HR-CTV contour was propagated from the FB-CT to the daily CBCT using mask-to-mask DIR via the PlatiPy library in Python (Chlap and Finnegan 2023) as follows:

(i) Obtain masks: The manual LR-CTV contour on the FB-CT was set as the moving mask and the nnUNet-generated LR-CTV contour on the CBCT was set as the fixed mask.
(ii) Affine registration: Linear affine registration between the moving and fixed masks was performed to initialise the registration.
(iii) Deformable registration: The ‘Fast symmetric forces demons’ registration algorithm in PlatiPy (Chlap and Finnegan 2023) with 8 cores, 1 stage (resolution down-sampled by a factor of 8), and 20 iterations was used to register the moving and fixed masks after affine registration initialisation.
(iv) Contour propagation: A composite deformation vector field (DVF) from the affine and deformable registrations performed in steps 2 and 3 was generated. The HR-CTV mask from the FB-CT was transformed according to this DVF onto the CBCT.
(v) Post-processing: The final HR-CTV was calculated as the intersection of the deformed HR-CTV mask with the nnUNet-derived LR-CTV mask.

#### 2.3.3. U-Seg accuracy assessment

The agreement between the U-Seg generated contours (U-Seg1 and U-Seg3) and manual contours was measured using the dice similarity coefficient (DSC).

A Wilcoxon Signed Rank test was performed on the DSC results for each structure to see whether incorporating patient-specific prior information into the network as additional input channels (U-Seg3) offered significant improvements in segmentation accuracy compared to the single-channel implementation (U-Seg1).

### 2.4. Development of qSOP

Using the cohort of 11 LACC patients who received POTD RT, we compared target and OAR coverage volumes between optimal and suboptimal plans to find (1) an appropriate structure and (2) corresponding cutoff values (in terms of per cent coverage) to devise a qSOP. These data were manually inspected to empirically derive appropriate thresholds for classifying plans as:

• Optimal: best balance of target coverage and OAR sparing as determined via offline audit.
• Acceptable: plans with adequate target coverage.
• Unacceptable: plans that do not satisfy target coverage constraints.

Thresholds were set by trial and error until the resulting qSOP could identify the optimal plan (as defined during offline audit by either observer) as frequently as possible. Particular attention was focussed on cases where: (1) multiple plans achieved full target coverage, (2) bowel sparing was *not* minimised, (3) there was a discrepancy between the plan delivered online and the optimal plan as judged by offline audit, and (4) no plan achieved full target coverage. For example, it was observed that the optimal plan did not always have 100% LR-CTV coverage, especially when substantial OAR sparing could be achieved, or when no plans providing that level of coverage were available. We compared accepted and rejected plans as a function of per cent LR-CTV coverage by each PTV for every fraction of every patient to find where the clinical decision boundary was located. This same analytical process was performed to assess acceptable levels of HR-CTV coverage and the tradeoff between bladder and bowel sparing. Examples of the analyses performed to devise the qSOP are elaborated in more detail in appendix E. In cases where inconsistencies were observed (i.e. observers rejected a plan providing 96% coverage in one fraction but accepted that level of coverage in another), the qSOP was designed to choose the more conservative threshold to avoid risking under-dosage of the target.

### 2.5. Automatic POTD selection tool

Based on the qSOP defined above, an automatic POTD assessment tool was developed. Given structure segmentations as inputs, this tool: (1) suggests the optimal plan, (2) highlights other candidate plans that also meet target coverage criteria, and (c) displays the amount of each structure covered by each PTV in an easily-interpretable table. The accuracy of the tool in identifying optimal and acceptable plans depends not only on the validity of the thresholds set in the qSOP, but also on the accuracy of the input structure segmentations. To disentangle these two effects, the frequency with which the tool could correctly identify the optimal plan (as defined by either observer during off-line auditing) was first calculated using the manual contours as inputs. Cases where a suboptimal but still acceptable plan was selected were also recorded. This analysis was repeated using U-Seg3 contours as inputs to the tool. The frequency with which optimal and acceptable plans were selected on-set was also assessed for benchmarking purposes.

## 3. Results

### 3.1. U-Seg3 performance

On an NVIDIA Quadro RTX 6000 GPU with 25GB of VRAM (CUDA version 11.4), the inference time of U-Seg3 was 1:40–1:45 (mm:ss) depending on the input image size. Broken down by step, the computation time was 1:30–1:35 and 10 s–14 s for nnUNet-segmentation and DIR, respectively.

**Figure 2.**
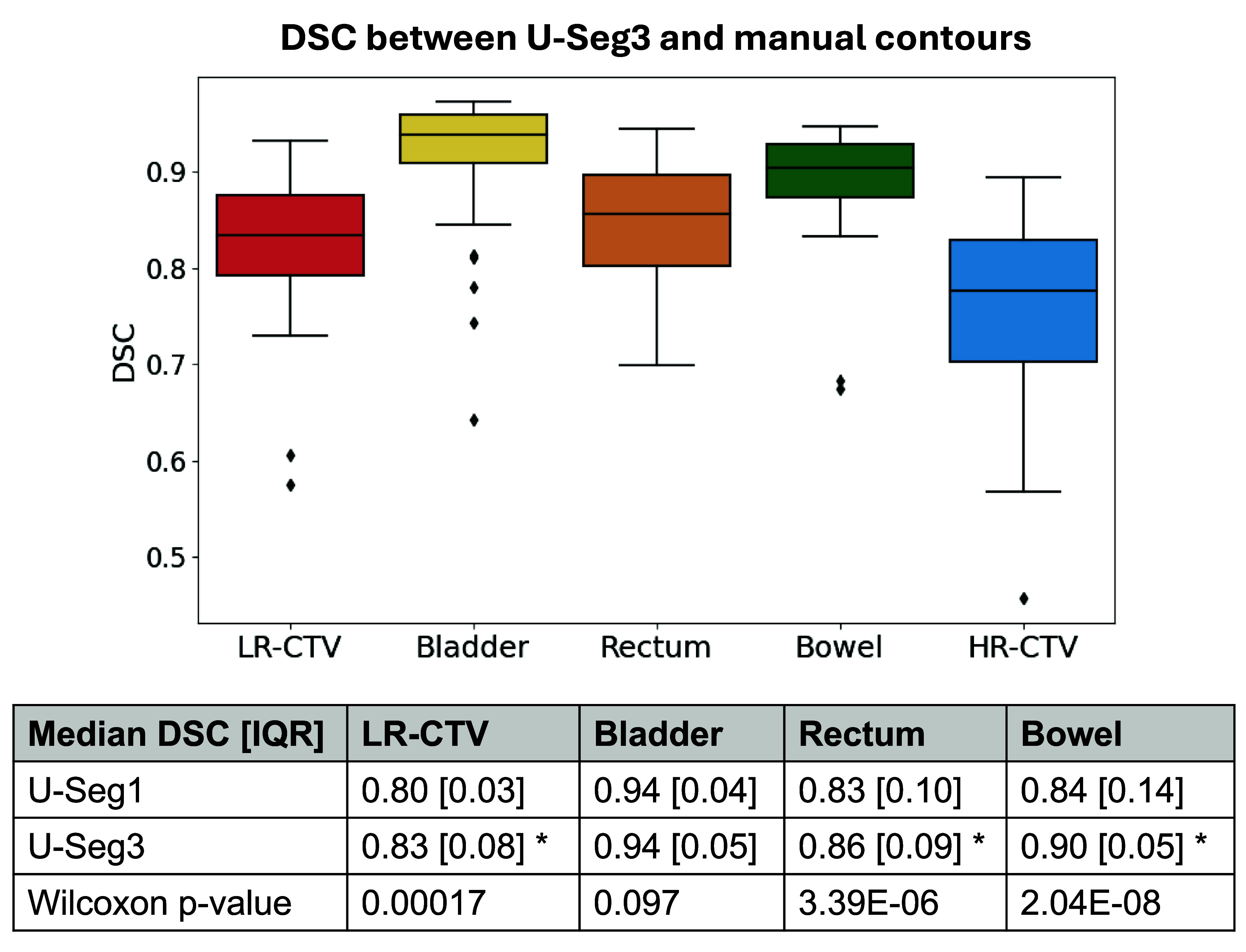
Top–Box and whisker plot showing the distribution of DSCs between U-Seg3-generated contours and manual contours for target and OAR structures in the test set of 8 patients (46 CBCTs). The height of the box corresponds to the interquartile range (IQR). The median is represented by the horizontal line in the centre of each box. The whiskers extend to data points that are not considered outliers. Outliers are denoted by the diamond symbol. Bottom–table comparing the median (IQR) DSC results obtained from the 1-channel and 3-channel input versions of U-Seg. Note that the 3-channel input had significantly better segmentation performance (*p* $ \ll $ 0.05) for all structures except the bladder.

**Figure 3.**
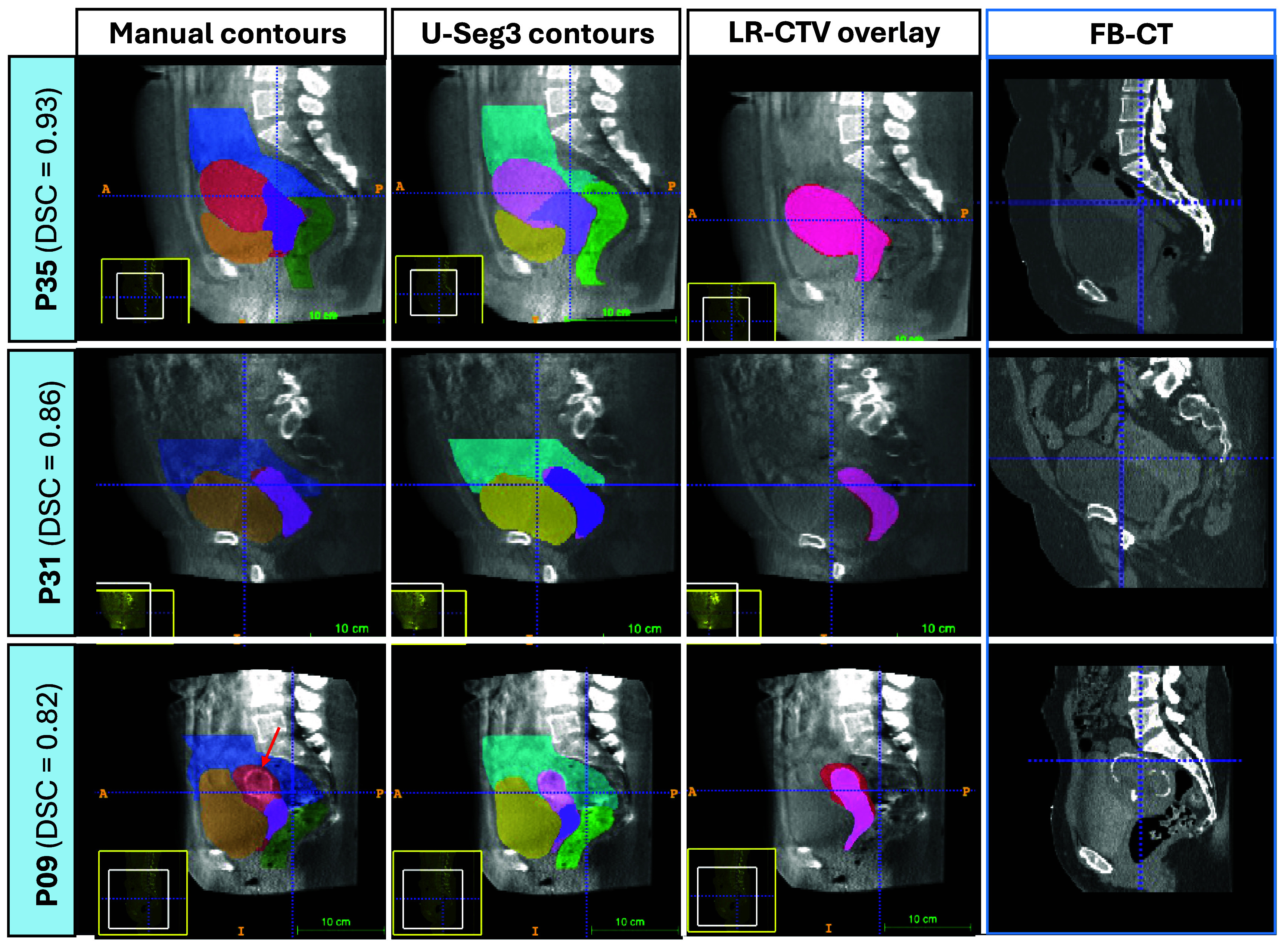
Example U-Seg3 segmentations (col 2) from 3 representative patients. The corresponding manual segmentations are shown in col 1. Col 3 shows the LR-CTV overlay between manual (red) and U-Seg3 (pink) LR-CTV contours. Col 4 shows the corresponding FB-CT that was used as an input to the nnUNet component of U-Seg3. Excellent segmentation performance is demonstrated even when the bladder and LR-CTV volumes are substantially different between treatment and planning (patient 35, row 1), and when the CBCT image quality is degraded by artefacts arising from a hip implant (patient 31, row 2). Patient 9 (row 3) demonstrates an example of U-Seg3 error, where the calcified fibroid (red arrow) was misinterpreted as the uterine boundary. Despite this error, the LR-CTV DSC was still good (0.82) and the optimal plan was still selected given this segmentation as input.

The median (interquartile range) DSC between manual contours and U-Seg3 generated contours is shown in figure 2. The Wilcoxon signed rank test indicated that the U-Seg3 had significantly better agreement with manual contours than U-Seg1 for the LR-CTV, rectum, and bowel bag. As the U-Seg3 was better, the U-Seg1 was not used to generate the HR-CTV or considered further in any aspect of this work. Examples of segmentation performance for 3 representative cases are given in figure 3.

### 3.2. Empirically-derived qSOP

**Figure 4.**
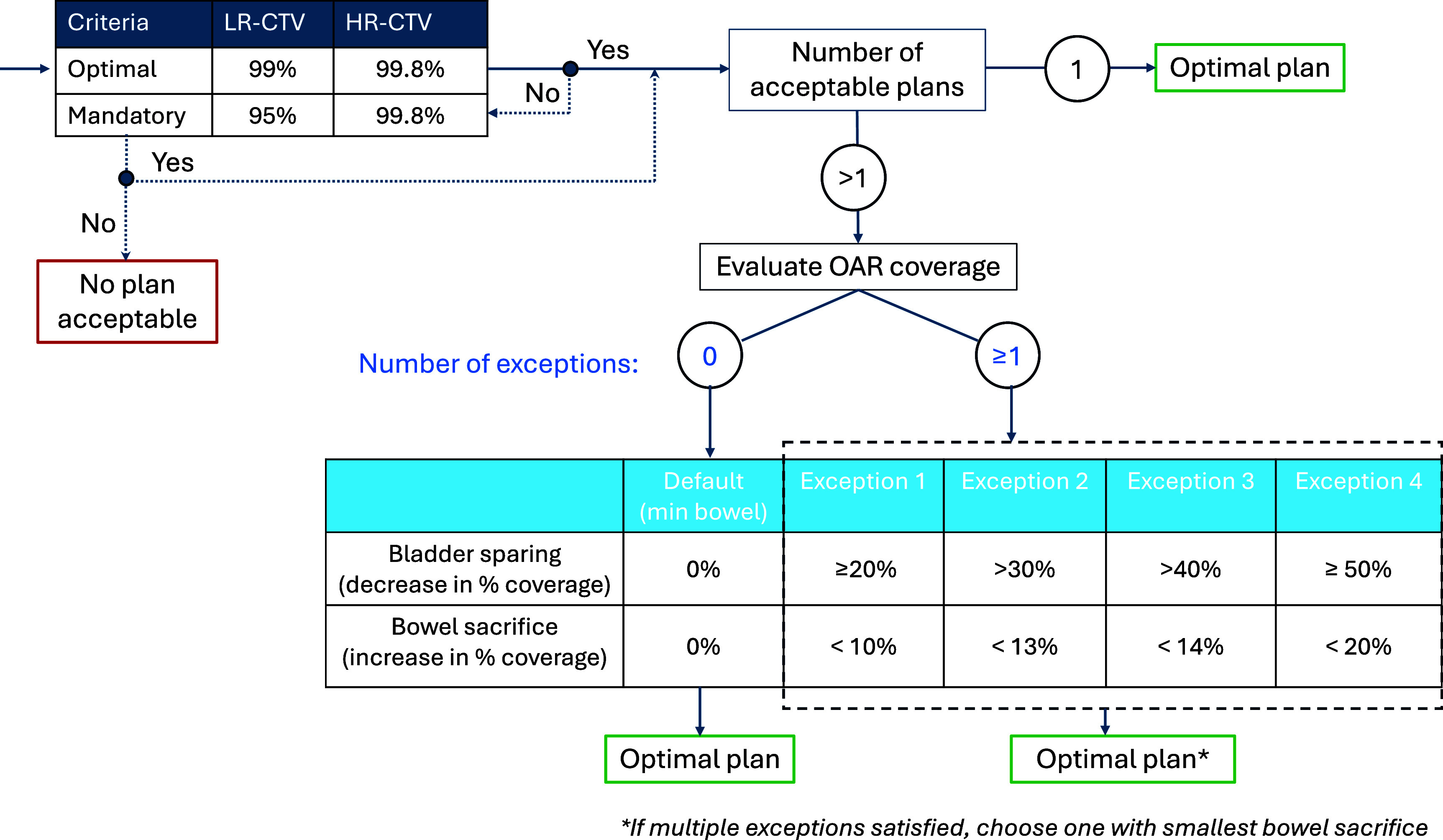
Quantitative SOP (qSOP) empirically derived from this work to maximise the frequency with which the optimal plan was selected based on target and OAR per cent coverage statistics.

The structure and thresholds of the empirically-derived qSOP are shown in figure 4. The qSOP first assesses target coverage: any plans providing optimal coverage criteria (99% and 99.8% for the LR-CTV and HR-CTV respectively) are considered treatment candidates. If **no** plan meets optimal target coverage criteria, then any plan satisfying mandatory coverage criteria (95% and 99.8% for the LR-CTV and HR-CTV respectively) is considered a treatment candidate. In the situation in which multiple plans are potential candidates, then the trade-off between bladder and bowel sparing is assessed. The optimal plan selected by default would be the candidate with the least amount of bowel coverage. However, exceptions can be made to allow a small sacrifice in bowel coverage in order to spare proportionally larger amounts of bladder. For example, Exception 1 indicates that a plan that increases bowel coverage by $ < $10% would be optimal if it provides $\unicode{x2A7E}$20% sparing of the bladder. The amount of allowable bowel sacrifice increases with increasing bladder sparing potential up to a maximum of 20% increased coverage, as shown in figure 4.

### 3.3. Performance of quantitative POTD assessment tool

**Figure 5.**
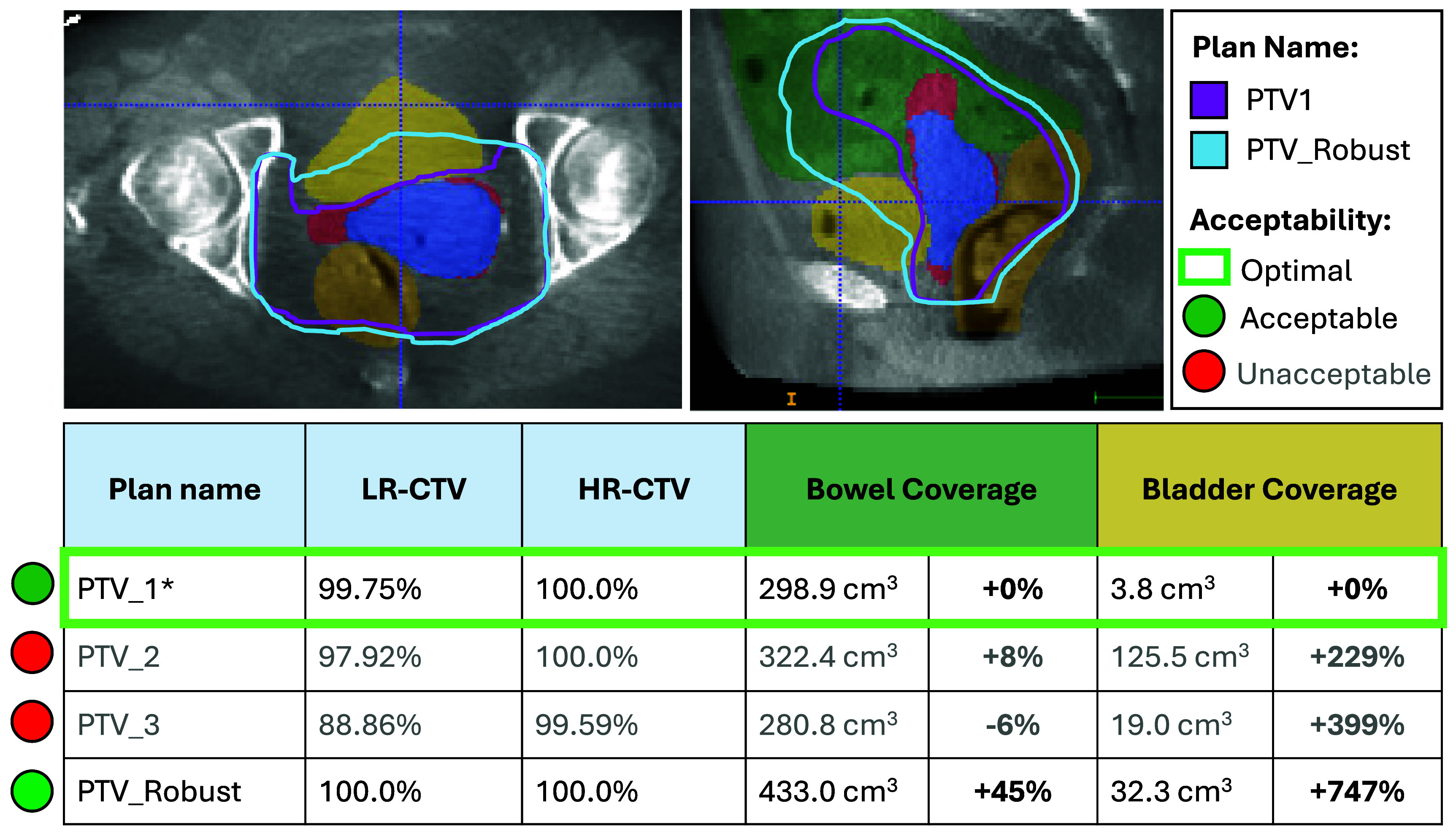
Mock-up of POTD assessment tool. All PTVs in the library and the underlying U-Seg3 segmentations are available for visual inspection on the 3D CBCT for transparency. (Note, only PTV1 and PTV_Robust are shown here for clarity). Target coverage is assessed according to the qSOP to flag plans as either ‘acceptable’ (green circles) or ‘unacceptable’ (red circles). The optimal plan indicated by the qSOP is highlighted by the green rectangle. Calculated values of LR-CTV, and HR-CTV coverage are given as a per cent for each PTV in the library. Similarly, the per cent difference in volumes for the bowel and bladder are also given (normalised to the acceptable plan with the minimum amount of bowel coverage).

A mock-up of the automatic POTD assessment tool is given in figure 5. In addition to highlighting the optimal plan, the tool indicates all plans that would be considered acceptable given calculated values of target coverage. For the sake of transparency, the resulting U-Seg segmentations and differences in per cent coverage of the OARs are also given to allow for quick on-set interpretation of the results.

#### 3.3.1. qSOP performance using ground truth manual contours

To assess qSOP performance independently of U-Seg3 auto-contouring, manual contours were used as inputs for the automatic POTD assessment tool. This led to optimal plan selection in 50/60 cases (83%). In 59/60 cases (98%), the selected plan was still deemed acceptable. Perfect classification was unattainable due to observer variability in determining allowable target miss and balancing bowel and bladder sparing, as the audit was based solely on visual inspection without contours and corresponding coverage statistics. The one failure case still showed 99.2% and 99.9% coverage of LR-CTV and HR-CTV, levels typically considered acceptable.

#### 3.3.2. qSOP performance using U-Seg3 contours

Given the U-Seg3 generated contours as inputs, the automatic POTD assessment tool selected the optimal plan in 46/60 cases (77%). In cases where the optimal plan was not correctly identified, the plan chosen was considered acceptable in 57/60 cases (95%). For reference, the plan selected and delivered by two radiation therapists was considered optimal in 46/60 cases (77%) and acceptable in 58/60 cases (97%). These results are summarised in table 1.

**Table 1.** Comparison of plan selection techniques in terms of agreement with offline audit. The qSOP with U-Seg3 contour inputs is the combination of every aspect of this work, and reflects the frequency with which the automatic plan assessment tool can identify optimal and acceptable plans. The other two plan selection SOPs are given for benchmarking purposes; the qSOP with manual ground truth contours shows the hypothetical performance of the qSOP in the absence of auto-segmentation error, and the on-set selections demonstrate the frequency with which the therapeutic radiographers delivered the optimal/acceptable plan in clinic using the non-qSOP.

Plan selection SOP	Optimal	Acceptable
qSOP (U-Seg3 contours)	46/60 (77%)	57/60 (95%)
qSOP (manual contours)	50/60 (83%)	59/60 (98%)
On-set selection	46/60 (77%)	58/60 (97%)

## 4. Discussion

### 4.1. Results interpretation

The incorporation of U-Seg3-generated contours into the novel qSOP derived in this work has enabled us to develop an automatic POTD assessment tool that can identify the optimal treatment plan for LACC patients with the same accuracy as a pair of human observers in clinical practice. This excellent outcome is largely due to the high performance of U-Seg3, which accurately segments all relevant target volumes and OARs on daily CBCT images. Not only is this accuracy in line with expected inter-observer contour agreement on CBCT images (Mason *et al* 2019), but it also outperforms previously published methods in terms of scope (*entire* target volumes, all OARs) and agreement with manual contours (see table 2). Indeed, U-Seg3 is robust across a large range of cancer stages (FIGO 1B2–4B), in the presence of extreme intra-patient variability in bladder and target volume (row 1 in figure 3), and in cases where CBCT image quality is degraded by artefacts arising from factors such as bowel gas and hip implants (row 2 in figure 3).

**Table 2.** Comparison of mean DSC results of U-Seg3 with other published approaches for CBCT-based adaptive RT for LACC patients.

Structure	Zhang *et al*	Beekman *et al*	U-Seg3
Training set:	[17–18 patients, 197–216 CBCTs]	[20 patients, 80 CBCTs]	[25 patients, 146 CBCTs]
Test set:	[5–6 patients, 56–73 CBCTs]	[10 patients, 20 CBCTs]	[8 patients, 46 CBCTs]

Partial LR-CTV (uterocervix only)	0.79	0.78	—
Full LR-CTV (parametria included)	—	—	0.82
HR-CTV	—	—	0.76
Bladder	0.84	—	0.92
Rectum	0.75	—	0.85
Bowel bag	0.81	—	0.89

We have demonstrated that including patient-specific information from planning into the segmentation algorithm as additional channels significantly improves segmentation accuracy (see figure 2), which is one likely reason why U-Seg3 outperformed the method described by Zhang *et al* (2022) despite using similar nnUNet-based network architectures.

#### 4.1.1. Impact of contouring errors on plan selection

Although U-Seg3 has an excellent performance overall, it failed to accurately segment the LR-CTV in 2 out of 5 fractions in the patient with an intrauterine device (see outliers in figure 2), which is unsurprising given that there were no similar cases in the training set. None of these segmentation errors resulted in the automatic selection of an unacceptable plan, though one resulted in sub-optimal plan selection.

Other anatomical features that occasionally caused U-Seg3 segmentation errors (albeit on a smaller scale) included retroverted uterus positions, calcified fibroids (see row 3 in figure 3) and gas bubbles in the uterus-cervix complex. These segmentation errors translated into unacceptable plan selections in 3 cases in total; 1 in the patient with large calcified fibroids and 2 in a patient who had both a consistently retroverted uterus *and* gas bubbles in the target volumes adjacent to the rectum. Although considered failure cases, there were no cases of gross target miss as shown in table 3. There were also no cases in which segmentation errors in the OARs were responsible for the selection of an unacceptable plan. Aside from the patient with the IUD as discussed above, the remaining three cases where a sub-optimal (but still acceptable) plan was selected can be traced back to cases where the border of the PTV coincided with the uterus-bladder border. Here, there were minor variations between U-Seg3 and manual contours (on the order of a few voxels) which was enough to push the qSOP towards different plan recommendations.

**Table 3.** Analysis of ‘failure’ cases where qSOP automatically selected an unacceptable plan due to U-Seg3 segmentation errors in the LR-CTV. In all 3 cases, the specific anatomy was under-represented in the dataset used to train U-Seg3. Note that although the plans automatically selected were not considered acceptable by expert review, the LR-CTV and HR-CTV target miss was less than 2% and 0.36% respectively.

Patient ID	Optimal plan (% coverage)		Automatically selected plan (% coverage)			
	LR-CTV	HR-CTV	LR-CTV	HR-CTV	Manual vs U-Seg3 DSC	Reason for failure
09 ($T\,\times\,0$)	100	100	98.3	100	0.76	Calcified fibroids (under-represented anatomy) and poor image quality. U-Seg3 under-segmented target at uterine fundus.

17 ($T\,\times\,3$)	100	100	99.51	99.64	0.73	Gas bubbles in uterocervix adjacent to rectum and retroverted uterus (under-represented anatomy). U-Seg3 under-segmented at posterior border of CTV.

17 ($T\,\times\,6$)	99.9	100	98.63	100	0.83	Gas bubbles in uterocervix adjacent to rectum and retroverted uterus (under-represented anatomy). U-Seg3 under-segmented parametria (left-right extent of CTV)

One advantage of POTD techniques is their robustness in the presence of some positional uncertainty. Indeed, the excellent success rate of our automatic POTD assessment tool highlights that even with minor contouring errors, automating adaptive RT is feasible using widely-available CBCT imaging technology.

#### 4.1.2. Impact of qSOP on plan selection

The other component of POTD selection is the SOP which is followed to classify plans as optimal, acceptable, or unacceptable. Here, we introduce the concept of a qSOP to showcase how the richer and more complex information afforded by segmentation could be incorporated into a user-friendly workflow. In this work, we retrospectively assessed the relationship between coverage statistics and off-line audit results to define thresholds for a SOP that would maximise classification accuracy of optimal plans. Even in the presence of observer inconsistencies in audit (particularly regarding the allowable amount of LR-CTV that could be missed by the PTV), our qSOP was able to identify the optimal plan in 83% of cases which is not only an improvement over what was achieved on-set, but also satisfies the 80% pass requirement used in observer training (Alexander *et al* 2019).

### 4.2. Study limitations

There are several limitations to this study and potential implementation barriers for this technology. Firstly, U-Seg3 performance could be improved by including more examples of patients with under-represented anatomy (i.e. retoverted uteruses, gas bubbles/large calcifications in the uterus) in the training set to further improve network generalisation and segmentation accuracy. Relatedly, all training and testing were conducted using CBCT images acquired via Eletka’s XVI system at a single institution, so it is unknown how the algorithm would perform on data acquired from other imaging systems or institutions.

It *is* possible to modify the network architecture, parameters, data augmentation steps etc if desired as the nnUNet code is all open source. Although the results from our current implementation were excellent, further gains (in terms of accuracy and processing speed) could potentially be made by tweaking the network or using techniques such as multi-threading in future versions.

Although the implementation of our automatic POTD selection tool could alleviate departmental resource burdens and improve the confidence with which an observer could make a selection (particularly at institutions not currently using POTD protocols), the hardware requirements needed to run the segmentation on a clinically relevant time scale need to be considered. This might require a dedicated GPU workstation to be used in-house, or setting up a data anonymisation and transfer pipeline to a web-based service to enable sufficiently fast computation.

### 4.3. Future work

Our next steps would be to give observers access to U-Seg3 at the time of auditing so that quantitative information regarding target and OAR coverage could be explicitly accounted for when defining the optimal plan. This would not only help resolve inconsistencies in fringe cases, but could also provide a means to refine the qSOP to improve convergence to a truer optimum. It would also be prudent to get clinician and therapeutic radiographer input on defining the thresholds and exceptions within the qSOP to ensure that the algorithm aligns with clinical experience and knowledge.

Although the pilot application of U-Seg3 has been in CBCT-based POTD selection, it could be fine-tuned to work in other modalities such as CT (which is of particular interest in tomotherapy systems) and/or adaptive RT workflows such as full online replanning. The latter would involve not only increasing the number and variability of patients in the training set, but would also likely harness advancements in CBCT technology such as polyenergetic quantitative reconstruction (Mason *et al* 2017) such that the underlying image quality is improved.

In the context of POTD selection, our ultimate aim would be to use U-Seg3 within the qSOP-based automatic plan assessment tool to improve the frequency with which the optimal plan is delivered whilst simultaneously alleviating departmental resources. We envision the possibility of this tool replacing *one* of the observers required on-set such that quality assurance and staff skill is maintained without overburdening RT departments. In our department we require 18/70 (26%) cervix-POTD trained radiation therapists to ensure smooth clinical implementation of this adaptive technique. This could be reduced to 9/70 (13%) if only one person was needed on-set to make the plan selection, which would significantly reduce hospital resources. Getting to this point would require further development and testing of the automatic plan assessment tool in observational studies, and a gradual roll-out in prospective clinical trials. Given the desire to keep therapeutic radiation therapists in the loop, we have intentionally designed the automatic plan assessment tool to be transparent such that the segmentations themselves can be visualised alongside all of the coverage statistics so that a human observer can easily interpret all of the results and make the ultimate treatment decision. This could enable the inclusion of more plans in the library generated (1) offline before treatment, (2) during the course of treatment offline in an ‘evolutive POTD’ approach (Rigaud *et al* 2018), and (3) online in specialised adaptive RT systems in a novel POTD-oART hybrid workflow (de Leon *et al* 2023).

Other potential benefits that could be quantified in future work include:

• Avoiding disruption to other units. As two cervix-POTD trained radiation therapists are not typically working on the same linear accelerator at the same time, one must be ‘borrowed’ from another unit to perform the plan selection process. This can be logistically challenging and time consuming.
• Providing plan recommendations based on a qSOP which has been empirically derived from treatment decisions made by experts. This could be particularly valuable for institutions who do not currently have any experience in cervix-POTD selection (which is the majority of centres worldwide), and could thus promote broader adoption of the POTD for LACC patients.

## 5. Conclusions

The vast majority of LACC patients are not treated with adaptive RT despite the proven dosimetric benefits. To address this critical gap in care, we have developed a segmentation-based automatic POTD assessment tool capable of identifying the optimal treatment plan with accuracy comparable to that of on-set observers. This tool offers quantitative data that brings transparency and standardisation to the plan selection process, potentially reducing the number of specially-trained observers required on-set while also lowering implementation barriers. Used in conjunction or independently, the U-Seg3 female pelvic segmentation algorithm and qSOP for automatic POTD selection could facilitate broader adoption of adaptive RT techniques to ultimately improve patient outcomes across healthcare settings.

## Figures and Tables

**Figure A1.**
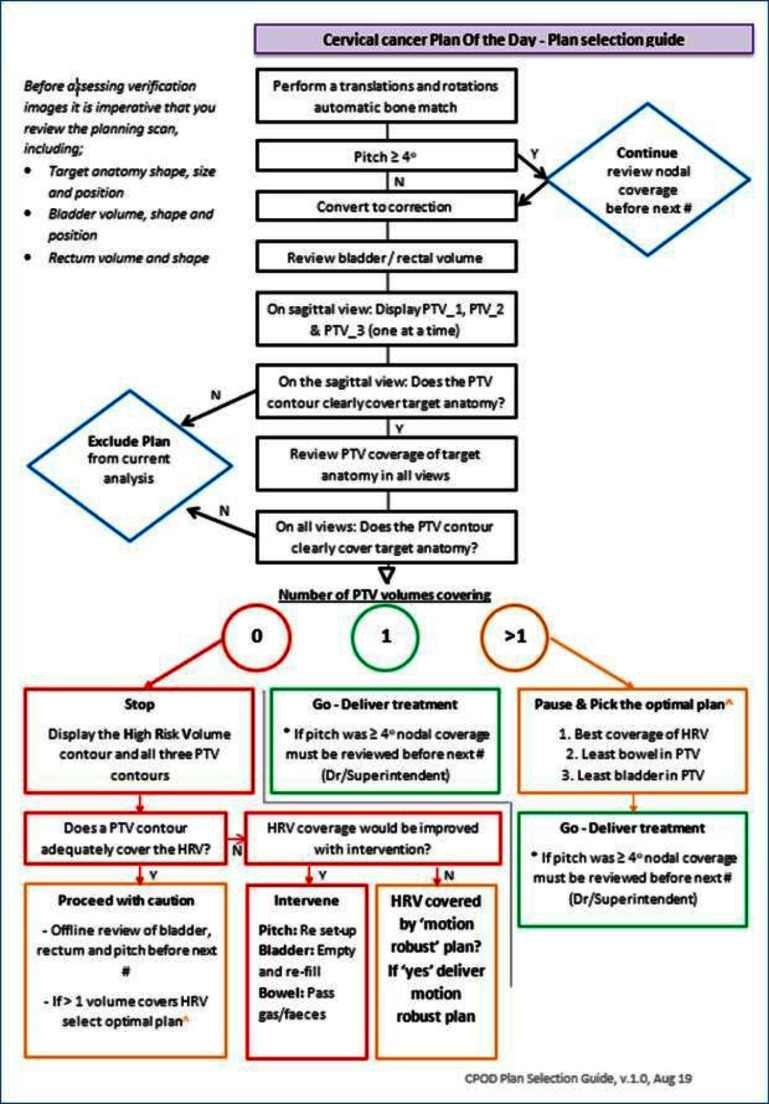
Standard operating procedure (SOP) used clinically at our institution to guide therapeutic radiographers to the optimal plan in POTD RT treatment for LACC patients.

**Figure B1.**
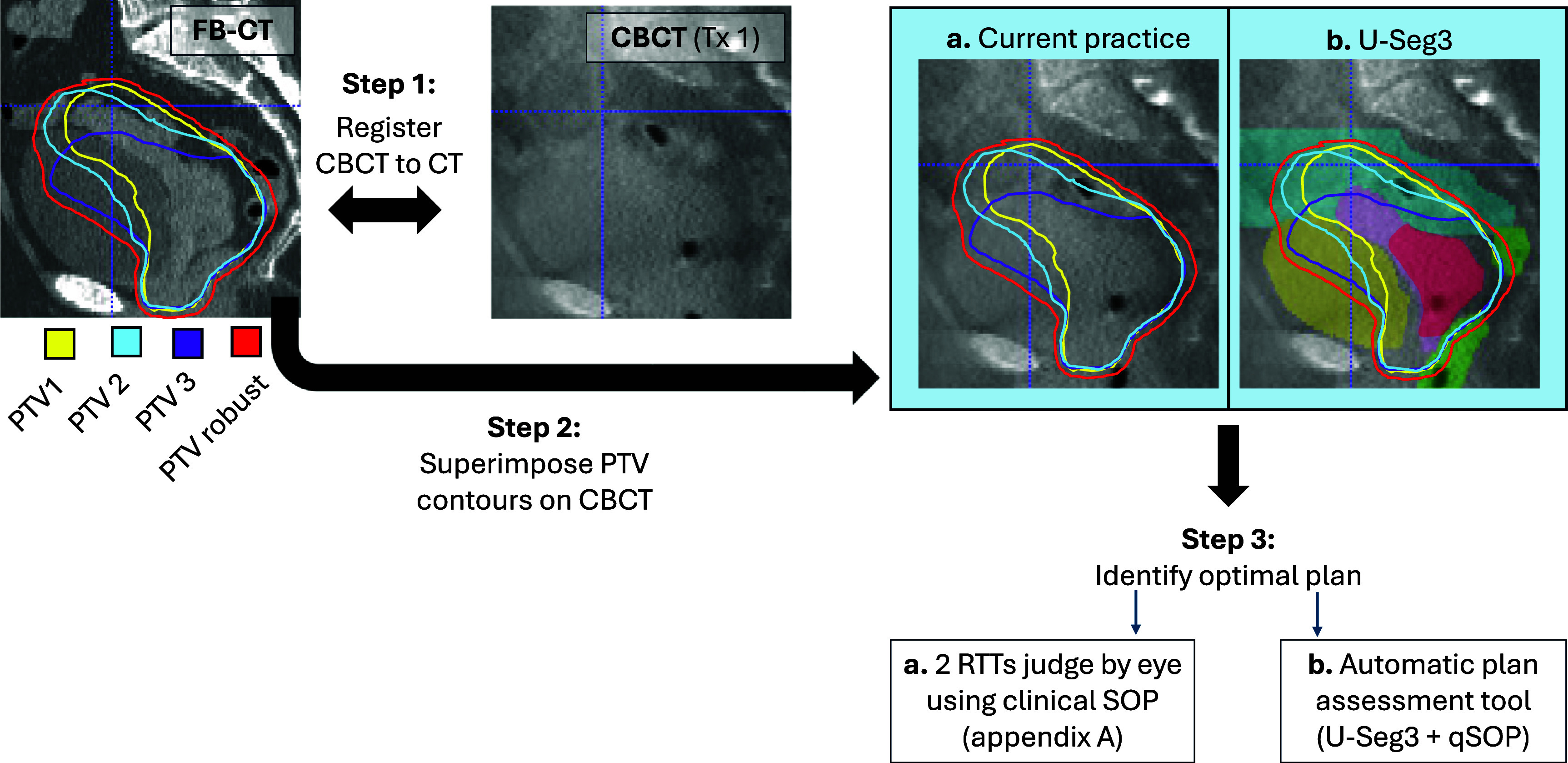
Indicates how planning target volumes (PTVs) were used for plan of the day (POTD) selection. Each plan in the library (plan 1, plan 2, plan 3, and plan robust) was designed using a different PTV to accommodate anticipated anatomical changes over the course of treatment (PTV1, PTV2, PTV3, and PTV robust). After standard CBCT–CT registration (step 1), each PTV was superimposed over the daily CBCT image (step 2). The optimal plan is considered to be the plan with the PTV fully enveloping the target structures whilst minimising the volume of healthy tissues (such as bladder and bowel) contained within. 3a: In current clinical practice, two specially trained RTTs visually inspect the image and identify the most optimal plan by eye. 3b: In the proposed work, the plan assessment tool U-Seg3 embedded within a novel qSOP automatically identifies the optimal plan given the calculated values of overlap between each structure of interest and each PTV.

**Figure C1.**
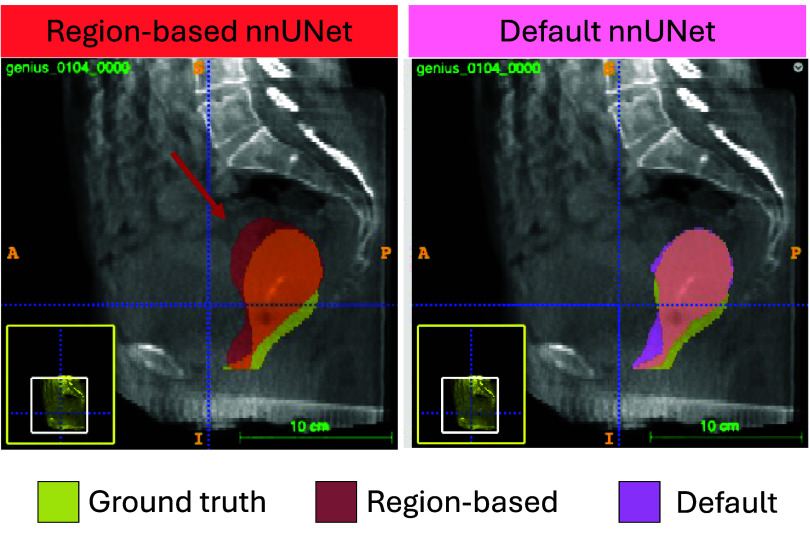
Comparison of LR-CTV segmentation results when using region-based feature in nnUNet vs using the default, non-hierarchical version of the nnUNet. Note that the region-based nnUNet has a poorer estimation of the uterine shape, particularly in the area denoted by the red arrow compared with the default nnUNet.

**Figure D1.**
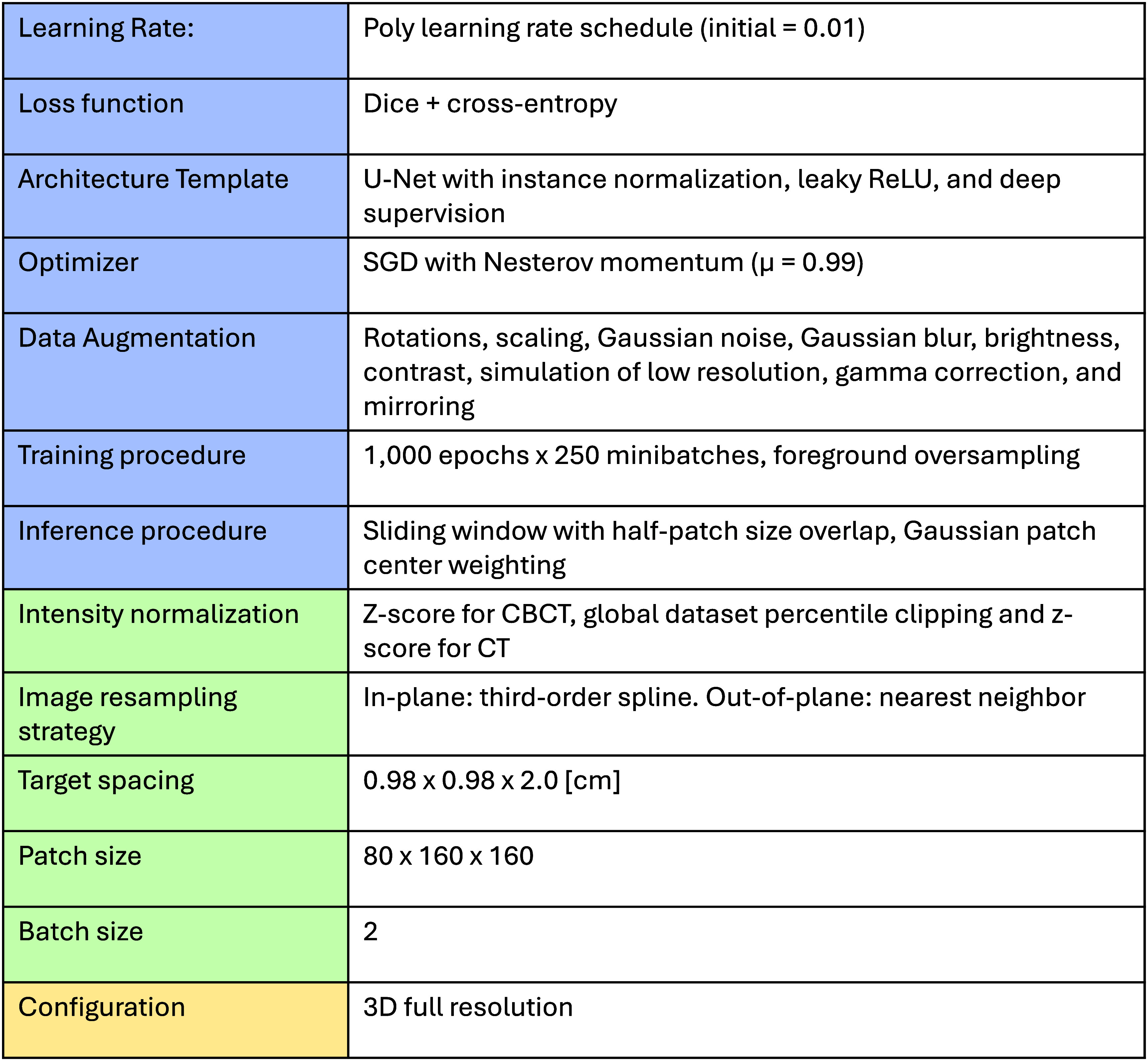
Describes nnUNet parameters where blue indicates nnUNet-fixed parameters, green indicates empirically-derived parameters, and yellow indicates parameters fixed by the authors of this work.

**Figure E1.**
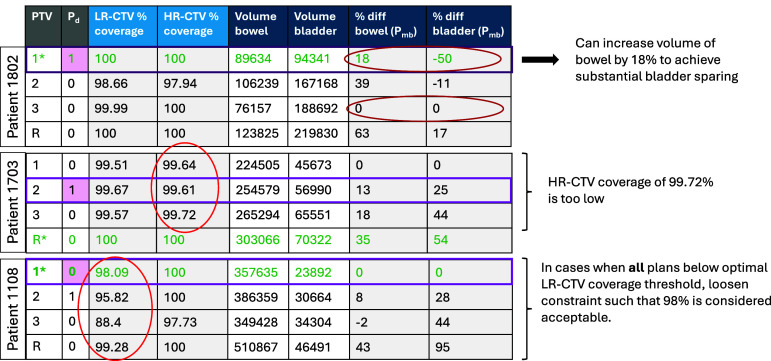
Indicates (cols 3-4) per cent coverage of each PTV in the library for target structures (LR- and HR- CTVs), (cols 5–6) the total volumes of bladder and bowel contained within each PTV (cc) and (cols 7–8) the per cent difference between bladder and bowel contained within the PTV normalised to the plan with the minimum amount of bowel coverage (that still had at least 98% LR-CTV coverage). The plan actually delivered on-set is denoted by a magenta rectangle and magenta shading in column 2. The optimal plan as determined via offline audit is written in green text and denoted by a * in column 1. Abbreviations: PTV = planning target volume, P_d_ = Delivered Plan, LR-CTV = low-risk clinical target volume, HR-CTV = high-risk clinical target volume, % diff = per cent difference, *P*_mb_ = plan irradiating minimum bowel (but still achieving at least 98% LR-CTV coverage).

## Data Availability

The data cannot be made publicly available upon publication because they contain sensitive personal information. The data that support the findings of this study are available upon reasonable request from the authors.

## Appendix C Rationale for 2-step process in U-Seg3 segmentation

We assessed the ability of the nnUNet to segment the HR-CTV as a sub-structure within the LR-CTV using the ‘region-based training’ feature so see whether it was worth attempting to combine all segmentation steps into a single U-Net architecture. Although the HR-CTV is not visible on CBCT, we hypothesised that the network might learn the spatial relationship between the LR-CTV and HR-CTV from the planning information given in the additional channels of U-Seg3. Although the combined LR-CTV and HR-CTV segmentation approach within the nnUNet was better than expected, the LR-CTV segmentation accuracy was slightly worse in the region-based version compared with the standard non-hierarchical version of the nnUNet (mean DSC in validation set of 0.828 vs 0.841). As this is the most important structure for determining an optimal plan, it would be prudent to choose an approach that maximises its segmentation accuracy. Figure C1 below gives a specific example of this.

We therefore decided to preserve the integrity of the LR-CTV structure segmentation by performing HR-CTV segmentation as a separate step leveraging the LR-CTV contour. Given the dependency of the HR-CTV segmentation on the LR-CTV segmentation, these steps must be run sequentially rather than in parallel, which necessitates the consideration of processing time. We therefore decided to use a fast implementation of DIR such that the HR-CTV position could be determined without dramatically increasing processing time or affecting the LR-CTV contour.

## Appendix E qSOP development

(i) **Patient 1802**: Although the original SOP (see appendix A) would guide users to choose PTV3, all observers agreed that PTV1 was optimal. This is due to the substantial amount of bladder sparing achieved (50% less bladder included in PTV1 compared with PTV3) despite an increase in the volume of bowel included in the PTV. To make the qSOP reflect cases like this in clinical practice, we devised ‘exception criteria’, which enable modest levels of bowel sacrifice that increase proportionally with the amount of bladder spared.
(ii) **Patient 1703**: This patient exemplifies how rejected plans were useful in determining quantitative target coverage criteria. All observers agreed that in this case, the only acceptable plan was PTV-Robust. LR-CTV coverage values alone were not enough to eliminate PTV1-3 (based on other case studies, including Patieint 1108 discussed below), which makes HR-CTV coverage the final arbitrator. From this example, we can see that HR-CTV coverage should be higher than 99.72% to be considered acceptable.
(iii) **Patient 1108:** This patient shows the decision-making process in cases where *no* plans provide full target coverage. In this case, the slight reduction in LR-CTV coverage (due to the PTV not completely covering the tip of the uterine fundus) from PTV1 was considered optimal given that HR-CTV coverage was still maintained, and much lower volumes of bowel and bladder were contained within the other candidate PTVs (which would be PTV2 and PTV-rRobust in this case). This was embedded in the qSOP by having optimal and mandatory levels of LR-CTV target coverage.
